# Carbon stock potential of highland bamboo plantations in northwestern Ethiopia

**DOI:** 10.1186/s13021-023-00224-2

**Published:** 2023-03-16

**Authors:** Ayana A. Jember, Mintesinot A. Taye, Getaneh Gebeyehu, Gashaw Mulu, Trinh Thang Long, Durai Jayaraman, Shiferaw Abebe

**Affiliations:** 1 Institute of Disaster Risk Management and Food Security Studies, Bahir Dar University, P. O. Box 5501, Bahir Dar, Ethiopia; 2 Department of Biology, Injibara University, Injibara, Ethiopia; 3 Department of Development Environment Management Studies, University of Gondar, Gondar, Ethiopia; 4 International Bamboo and Rattan Organization, Beijing, 100102 China; 5 Department of Geography and Environmental Studies, Assosa University, Assosa, Ethiopia; 6 Office of the Research Directorate, Assosa University, P. O. Box 18, Assosa, Ethiopia

**Keywords:** Biomass, Carbon stock, Ethiopia, Homestead, Riverbank, Woodlot, *Oldeania alpina*

## Abstract

**Background:**

In Ethiopia, highland bamboo has been cultivated in various niches: farmlands, riverbanks, woodlot boundaries, and homesteads, and agroforestry systems. However, the biomass and carbon storage of potential of bamboo forests across niches is not well characterized in Ethiopia. Therefore, this study was conducted to estimate the biomass and carbon storage potential of highland bamboo plantations in northwestern Ethiopia. To this end, a total of 60 circular plots measuring 100 m^2^ with a radius of 5.64 m were randomly established on the homestead, woodlot, and riverbank plantation niches to conduct the inventory. The biomass storage of bamboo was calculated based on previously published allometric equations. Biomass and carbon stock variations among age-classes and niches of bamboo forests were analyzed using analysis of variance (ANOVA) and subsequent pairwise means comparisons of carbon stocks among niches were performed via post hoc Tukey test at p < 0.05.

**Results:**

Results showed that the mean aboveground biomass (AGB) ranged from 150.18 – 191.42 Mg ha^−1^ in the entire niches. The highest amount of AGB was stored in the homestead niche (191.42 Mg ha^−1^) followed by the woodlot (180.11 Mg ha^−1^) and riverbank niche (150.17 Mg ha^−1^), respectively. The highest carbon stock (111.56 Mg C ha^−1^) was found in the homestead niche while the smallest amount was recorded in the riverbank niche (87.52 Mg ha^−1^). The homestead bamboo plantation has the highest biomass storage due to the application of manure and natural fertilizer, regular harvesting and management of culms, and protection from illegal harvesting and grazing.

**Conclusion:**

This study highlights the importance of bamboo plantations in climate change mitigation. Hence, bamboo plantation should be promoted; and natural resource management and forestry departments of the government, Universities, research centers, the International Bamboo and Rattan Organization (INBAR), and other partners should work with local communities to expand bamboo plantation on their homesteads and degraded lands.

**Supplementary Information:**

The online version contains supplementary material available at 10.1186/s13021-023-00224-2.

## Background

Today, climate change is one of the most pressing challenges of humanity. Consequently, scientists, resource managers, and policymakers have paid utmost attention to climate change mitigation [1, 2]. In this regard, carbon (C) sequestration and storage in forest ecosystems has been identified as the vital mitigation strategy for the changing climate [3–5]. Second, only to the oceans, forests play an essential role in the C cycle, accounting for a more significant proportion of C exchange between the atmosphere and terrestrial biosphere than other land biomes, thereby contributing to climate change mitigation [6, 7]. Forest ecosystems store more than 80% of all terrestrial aboveground C and more than 70% of all soil organic carbon [7, 8].

Bamboos are important vegetation resources that are mostly found in tropical and sub-tropical regions of Asia, Africa, and Latin America [9–11], covering 35 million hectares globally [12] and accounting for about 1.0 percent of the world’s forest area [13]. Because of their rapid growth and ease of propagation, bamboos are unique in their ability to meet a wide range of ecosystem services [1, 2, 14–16]. Bamboo is one of the fastest-growing plants [17], with daily growth rates ranging from 30 to 100 cm and harvesting cycles ranging from 3 to 5 years, compared to 10–50 years for most timber species [18–21]. Plants sequester CO_2_ during photosynthesis, playing an important role in climate regulation. In this regard, bamboo has a unique capacity to absorb CO_2_ from the atmosphere and functions as an important C sink [22–25]. Bamboo forests have been found to have higher C storage per hectare than fast-growing tropical and sub-tropical trees under comparable conditions due to their rapid growth and short harvesting cycle [21, 25–27].

In recent decades, bamboo has become a globally important biomass resources in many parts of the world [5, 28, 29]. Bamboo's recent recognition into volunteer carbon finance mechanisms has increased its attractiveness as a plantation species [30]. Bamboo forests can be planted in degraded tropical forests because they are an important component of most tropical forest ecosystems and are adaptable to adverse site conditions [1, 31]. While contributing to environmental sustainability, they also provide income as well as a range of goods and ecosystem services for rural households, thus, contributing to food security and poverty eradication [32]. Bamboo can be grown in agroforestry systems on agricultural lands and farms, in rural landscapes, and along roads, rivers, human settlements, and trees in and around cities [33]. Bamboo-based agroforestry has the potential to improve production, sustainability, and resource conservation [34]. Many useful bamboo species may coexist with trees in the same biological niche, making them suitable for agroforestry [33, 35, 36]. Bamboo outperforms most tree species due to its fast growth. Bamboo, for example, grows three times faster than eucalyptus and matures in just 3 years, so large-scale efforts to promote bamboos in agroforestry systems are underway [35].

Ethiopia owns about 67% of Africa’s and 7% of the world’s total bamboo forest areas, making it one of Africa’s largest bamboo resource bases [37–39]. The country has two indigenous bamboo species: the lowland bamboo, *Oxytenanthera abyssinica* (A. Richard) Munro, and the highland bamboo, *Oldeania* or *Yushania alpina* (K. Schumann) Lin, with an area coverage of more than one million ha [37]. These species are indigenous to Ethiopia and endemic to Africa and are only found in the sub-Saharan region [40]. Unlike lowland bamboo, highland bamboo is commonly cultivated and managed by farmers in various agroforestry systems [41]. It grows in different niches, including farmland patches, riverbanks, farm boundaries, roadsides, homesteads, and even urban areas at an elevation ranging from 2,200 to 4,000 m [42].

A thorough understanding of carbon storage patterns in forest ecosystems is crucial for forest management to slow the rate of climate change [28]. The distribution of carbon stored in plant biomass and the rate of carbon sequestration within forest landscapes can be variable at local scales [43]. Because specific niches or environmental surroundings affect plants’ growth and performance [44, 45]. Local forest carbon dynamics are related to biotic and abiotic productivity drivers such as species composition and forest diversity, as well as abiotic productivity drivers such as soil type, topographic position, aspect, and elevation [28, 46, 47]. However, despite the fact that bamboo-based agroforestry has been practiced in different plantation niches, the biomass and carbon storage of potential of bamboo across niches is not well characterized in Ethiopia. Indeed, some researches on the productivity and stand structure of highland bamboo along plantation niches has previously been undertaken [48, 49]. However, they concentrated on the productivity of bamboo in niches while overlooking its function in carbon sequestration and thus climate change mitigation. With this backdrop in mind, this study was conducted to estimate the biomass and carbon storage potential of highland bamboo (*Oldeania alpina)* forests over plantation niches in northwestern Ethiopia.

## Materials and methods

### Description of the study area

**Fig. 1:**
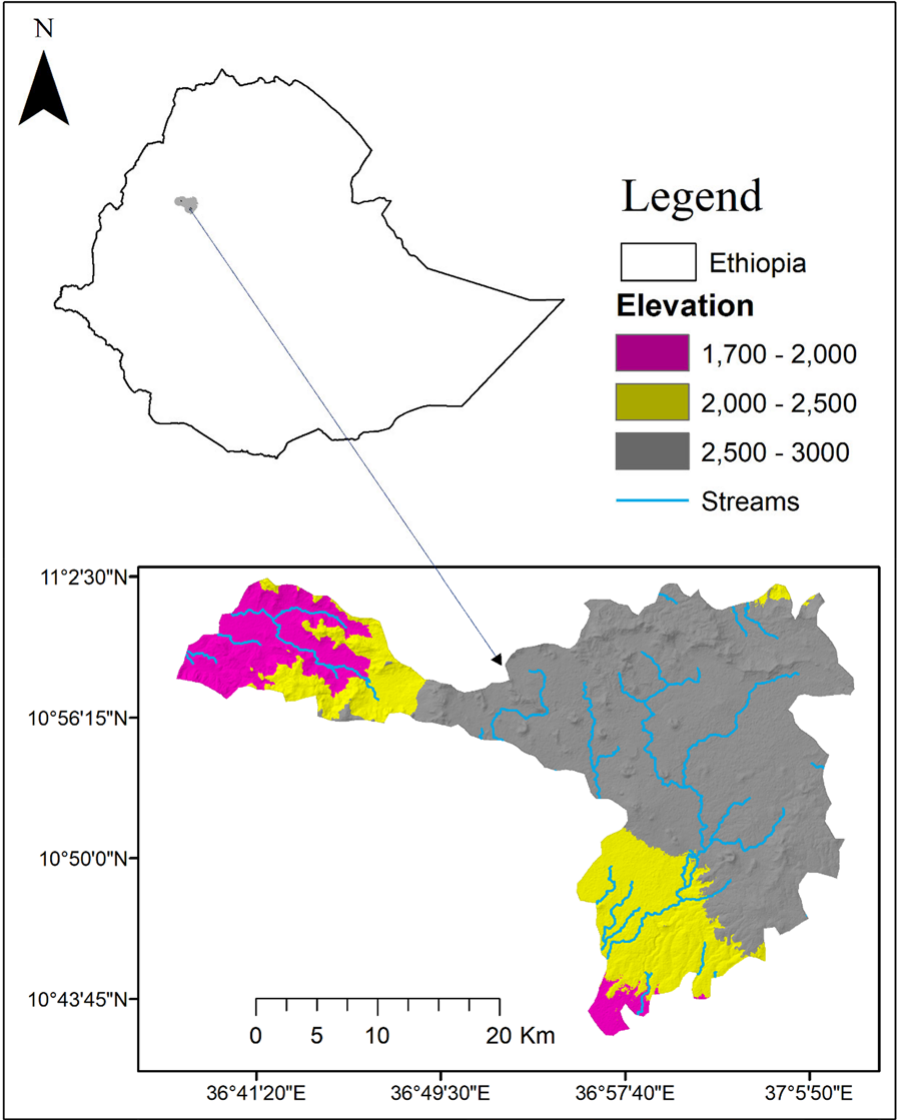
Map of the study area

The study was conducted in Banja and Guagusa-shikudad districts of northwestern Ethiopia. The study districts are located between 10° 43′ 45'' to 11° 2′ 30'' N and 36° 41′ 20'' to 37° 5′ 50'' E, with a total area of 7964.5 km^2^. The districts are situated 490 km from Addis Ababa and are parts of the northwestern highlands of Ethiopia (Fig. 1). The elevation ranges between 1700 and 3000 m. The topography is typical of volcanic landscapes, with volcanic rocks deeply incised by streams, resulting in the current ragged and undulating landforms.

The soils of the study areas are primarily formed from volcanic parent materials that are reddish or brown, drain freely, and have a medium to heavy texture [50]. Andisols, nitosols, and cambisols are the three major soil types [51]. There are both perennial and seasonal rivers and streams that flow over the study districts. According to National Meteorology Agency climate data, the districts receive the majority of their rainfall during the summer season (June to September) and receive approximately 2300 mm of mean annual rainfall while the mean annual temperature ranged between 11 and 24 °C. Like most Ethiopian highlands, the major land use-land covers of the study area are agricultural land, grazing/pasture land, forest, and settlement.

The study area contains a variety of vegetation resources that are classified as dry Afromontane forests [52]. *Albizia gummifera, Croton macrostachyus, Prunus africana*, and *Apodytes dimidiata* are the dominant species. In elevation zones 1850–2100 m, *Albizia gummifera* and *C. macrostachyus* dominate. *P. africana* and *A. dimidiata* found between 2100 and 2350 m, while *Juniperus procera* and *Ekebergia capensis* found sparsely with undergrowth shrub species at higher altitudes [53]. The hallow-stemmed Afro-alpine or mountain bamboo [*Yushania alpina or Oldeania alpina* (K. Schum.) W.C. Lin], locally known as *Kerkha*, is one of the main vegetation types grown in the study area [54]. It is a monopodial/leptomorphic rhizome bamboo species growing in the south, southwest, central, and northwestern highlands of Ethiopia [38].

Farmers in the study area grow and cultivate highland bamboo as an agroforestry system in various niches such as homesteads, woodlots, riverbanks, farm boundaries, and roadsides. They harvest bamboo culms for a variety of socioeconomic uses, including a source of income, furniture, household utensils, and so on. The bamboo plantations in the homestead niche are relatively well-managed, with the use of manure and natural fertilizer, proper harvesting, and protection from illegal harvesting and livestock. Bamboo culms in the woodlot, roadside, and riverbank niches, on the other hand, are subjected to illegal harvesting and free grazing, resulting in decreased culm density and productivity.

### Sampling and data collection techniques

The four bamboo growing sites (villages) namely, Gashana-akayeta, Kesa-chewsa, Jibayeta, and Ageza-garda, were selected purposively for the inventory based on their forest coverage and accessibility. A total of 60 circular plots (5 plots × 3 niches × 4 sites) measuring 100 m^2^ with a radius of 5.64 m were randomly established on the homestead, woodlot, and riverbank plantation niches to carry out the inventory. Circular plots are more efficient than square or rectangular plots because the actual perimeter of the plot is smaller; thus, the number of bamboo culms on the edge is limited [55]. A total of 20 culms were selected at random from each plot for each age group to collect stand structure and biomass data in bamboo forests (Additional file 1). In each plot, the diameter at breast height (DBH) and height (H) were measured, and the bamboo age (A) was determined using its morphological features [56, 57]. Culms in each plot were classified and counted based on their ages. A caliper was used to measure culm’s diameter at 1.3 m height, and a graduated stick (bamboo culm marked at 1 m intervals) was used to measure culm’s height.

### Stand structure and biomass carbon stocks estimation

The stand density of the highland bamboo was calculated following as follows [55]:

$$\mathrm{Density\,of\,culms ha}-1 = \frac{ \mathrm{Number\,of\,culm }*\mathrm{10,000}}{\mathrm{Plot area }({m}^{2})}$$

Likewise, the basal area was calculated as:

Basal area (BA) = $\frac{\pi *{DBH}^{2}}{4}$ Where, DBH is the diameter at the breast height of culms and π (pi) = 3.14.

The aboveground biomass (AGB) storage of highland bamboo forests was estimated using an allometric model developed by Abebe et al. [58] in the Masha Forest of Southwestern Ethiopia. The model was developed by harvesting randomly selected 1–2, 3–4, and 5–6-year-old culms of highland bamboo in Ethiopia. A total of 42 culms were harvested from seven circular plots (100 m^2^) based on age and diameter. The mean DBH of the sampled culms was 5.68 (1–2 year), 5.41 (3–4 year), and 5.09 for 5–6-year-old culms. The model has a robust predictive power with the coefficient of determination (adj. R^2^) in the range between 0.856 and 0.925. In this model, the relationship between AGB and DBH was predicted by a simple allometric function:

$$\mathrm{Y}=\mathrm{ a x }{\mathrm{DBH}}^{\mathrm{b}}$$

where, Y $=$ aboveground biomass and *a* and *b* are parameters.

$${\text{AGB}}1{ }\left( {1{ }{-}{ }2{\text{ years}}} \right) = 0.259{\text{ x DBH}}^{2.098}$$

$${\text{AGB}}2{ }\left( {3{ }{-}{ }4{\text{ years}}} \right) = 0.129{\text{ x DBH}}^{2.577}$$

$${\text{AGB}}3{ }\left( {5{ }{-}{ }6{\text{ years}}} \right) = 0.165{\text{ x DBH}}^{2.237}$$

Then, the total aboveground biomass (TAGB) was calculated by summing up of aboveground biomass of each age class as follows: $\mathrm{TAGB}={\mathrm{AGB}}_{1}+{\mathrm{AGB}}_{2}+{\mathrm{AGB}}_{3}$. The aboveground (AGB) to belowground biomass (BGB) ratio of 4:1 (0.25) [59] was used to estimate the BGB. The BGB was then computed as follows: $\mathrm{BGB}=\mathrm{AGB x }0.25$. Then, total belowground biomass $\mathrm{TBGB}={\mathrm{BGB}}_{1}+{\mathrm{BGB}}_{2}+{\mathrm{BGB}}_{3}$. Finally, the C storage and CO_2_ sequestration potential of the highland bamboo bamboo forests were calculated from total biomass (TB) (total aboveground and belowground biomass), respectively, as follows [60]: $\mathrm{Carbon }\left(\mathrm{C}\right)=\mathrm{C fraction }\left(0.47\right)\mathrm{ x TB};\mathrm{and }{\mathrm{CO}}_{2}=\mathrm{C x }3.67$.

### Statistical analysis

Data were organized in an excel spreadsheet and univariate statistics were calculated to determine stand structure and biomass among three niches of bamboo forests (Additional file 1). The normality of data distribution was checked using Shapiro–Wilk Test before analysis. Biomass and carbon stocks variations among age classes and niches of bamboo forests were analyzed using analysis of variance (ANOVA) in R statistical software and subsequent pairwise comparisons of means of biomass and carbon storage among niches of bamboo forests were performed via post hoc Tukey test at p < 0.05.

## Results

### Stand structure of the highland bamboo forests in the study area

The culm density of the highland bamboo stand showed significant variations across plantation niches. The highest stand density was recorded in the homestead (27,945 culms ha^−1^) followed by woodlot (22,775 culms ha^−1^) and riverbank (20,375 culms ha^−1^) niches. Like culm density, there was variation in the clump stocking among bamboo niches. The clump stocking in the stands ranged from 1,562 to 1,885 clumps ha^−1^. The woodlot represented the highest clump stock (1,885 clumps ha^−1^), while the homestead shared the smallest clump density (1,562 clumps ha^−1^). The clump stock was inversely proportional to the culm density. For example, the highest culm density (22,775 culms ha^−1^), but the lowest clump stock (1,562 clumps ha^−1^) was observed in the homestead niche. In terms of culm age composition, 3–4-year-old culms were the most common, while 5–6-year-old culms represent the smallest proportion in all plantation niches (Table 1).

**Table 1:** Stand structure of highland bamboo forests of the study area

Age (year)		Homestead					River basin					Woodlot			
	Culm (ha^_1^)	Clump (ha^_1^)	DBH (Cm)	BA (m^2^ ha^−1^)	Height (m)	Culm (ha^_1^)	Clump (ha^_1^)	DBH (Cm)	BA (m^2^ ha^−1^)	Height (m)	Culm (ha^_1^)	Clump (ha^_1^)	DBH (Cm)	BA (m^2^ ha^−1^)	Height (m)
1–2	8,750 ± 59	1,562 ± 11	5.96 ± 0.09	28.05 ± 0.90	14.48 ± 0.6	7,810 ± 65	1,775 ± 27	5.55 ± 0.11	24.46 ± 0.98	12.23 ± 0.53	8,915 ± 92	1,885 ± 46	5.78 ± 0.07	26.33 ± 0.66	14.12 ± 0.58
3–4	11,540 ± 88		5.54 ± 0.1	24.32 ± 0.91	13.56 ± 0.6	11,360 ± 81		5.04 ± 0.12	20.19 ± 0.92	11.56 ± 0.57	10,755 ± 80		5.39 ± 0.06	22.93 ± 0.55	13.12 ± 0.55
5–6	4,187 ± 85		4.82 ± 0.12	18.49 ± 0.95	10.4 ± 0.3	3,760 ± 11		4.45 ± 0.14	15.89 ± 1.00	9.11 ± 0.44	3,955 ± 31		4.74 ± 0.08	17.79 ± 0.58	10.14 ± 0.43
Total	27,945		5.44 ± 0.08	70.86^a^	12.81 ± 0.45	20,375		5.01 ± 0.09	60.54^b^	11.47 ± 0.34	22,775		5.3 ± 0.06	67.05^a^	10.97 ± 0.34

The 1–2-year-old culms had the highest thickness (DBH) in all niches. In contrast, 5–6-year-old culms had the lowest DBH value. The bamboo forests of the homestead niche had the thickest culms (6 cm), while the thinnest (5.5 cm) was observed in the river basin bamboo forest (Table 1). During field observations, it was understood that the predominance of the thickest bamboo culms over the homestead niche was associated with the application of natural fertilizers like cow dung and mulching. Regarding, the total basal area (BA), the homestead niche (70.86 m^2^ ha^−1^) covers a maximum area followed by woodlot (67.05 m^2^ ha^−1^) and river basin niches (60.54 m^2^ ha^−1^) in the bamboo forests. The fastest-growing nature of the highland bamboo revealed that a total basal area achieved a 73.5% of size increment at the age class of 1–2 and 3–4 years old from the entire growth life in the three niches. The analysis of variance (ANOVA) showed that there existed a significant difference between the total basal area of the homestead and river basin niches at p < 0.05 (Table 1).

### Variations of aboveground biomass of the highland bamboo at age classes

The pairwise mean AGB comparisons via post hoc Tukey test exhibited significant differences among the age class of bamboo in homestead and river basin niches at p < 0.05. In contrast, there was no significant difference in aboveground biomass of 1– 2-year-old (AGB1) bamboo culms among niches. The aboveground biomass stored by 5*–*6-year-old (AGB3) was lower than 1*–*2 and 3*–*4-year-old bamboo culms age of the highland bamboo in the three niches and showed significant difference at p < 0.05. The highest aboveground biomass was found in the 3*–*4-year-old bamboo culms of the plantation niches (Table 2).

**Table 2:** Tukey's pairwise comparisons of AGB among age class of highland among plantation niches

	HSAGB1	HSAGB2	HSAGB3	RBAGB1	RBAGB2	RBAGB3	WLAGB1	WLAGB2	WLAGB3
HSAGB1		0.9689	0.000117	0.0348	0.01354	1.03E-05	0.8856	1	2.85E-05
HSAGB2	1.606		1.04E-05	0.000473	0.000152	1.03E-05	0.2001	0.9284	1.03E-05
HSAGB3	6.667	8.273		0.8589	0.9548	0.0349	0.02863	0.000224	1
RBAGB1	4.554	6.161	2.113		1	0.000118	0.6902	0.05908	0.691
RBAGB2	4.957	6.563	1.71	0.4028		0.00035	0.492	0.02452	0.855
RBAGB3	11.22	12.83	4.553	6.666	6.263		1.03E-05	1.03E-05	0.0825
WLAGB1	2.026	3.632	4.641	2.529	2.932	9.194		0.9435	0.01057
WLAGB2	0.248	1.854	6.419	4.306	4.709	10.97	1.777		5.56E-05
WLAGB3	7.081	8.688	0.4145	2.527	2.124	4.138	5.056	6.833	

Number 1, 2 and 3 refer to age class 1–2, 3–4, and 5–6, respectively

*AGB* aboveground across niches, *RB* Riverine bamboo, *WL* woodlots and *HS* Homestead

### Variations in total biomass of the highland bamboo forests across plantation niches

The total aboveground biomass ranged from 150.18 to 191.42 Mg ha^−1^ in the entire niches. The highest amount of AGB was stored in the homestead niche (191.42 Mg ha^−1^) followed by the woodlot (180.11 Mg ha^−1^) and riverbank niche (150.17 Mg ha^−1^), respectively. The total aboveground biomass stored between the homestead and the woodlot didn’t show a significant difference (p < 0.05). However, the total aboveground biomass accumulated between the homestead and river bank niches; and woodlot and river bank niches showed a significant difference (p < 0.05) (Table 3).

**Table 3:** Analysis of variance (ANOVA) of AGB of highland among plantation niches and age groups

Niche	Biomass storage				
	AGB1 (Mg ha^−1^)	AGB2 (Mg ha^−1^)	AGB3(Mgha^−1^)	TAGB (Mg ha^−1^)	TBGB (Mg ha^−1^)
RB	56.26 ± 2.49	55.20 ± 3.54	38.72 ± 2.71	150.18 ± 7.58^b^	36.04 ± 1.82
WL	62.92 ± 1.78	67.59 ± 1.88	49.61 ± 2.07	180.12 ± 4.29^a^	43.23 ± 1.03
HS	68.25 ± 2.43	72.47 ± 3.29	50.70 ± 2.92	191.42 ± 7.63^a^	45.94 ± 1.83

Different letter indicates significance difference while similar letter showed no significant difference of biomass among niches at p < 0.05

Number 1, 2 and 3 refer to age class 1–2, 3–4, and 5–6, respectively

*AGB* aboveground biomass, *TAGB* Total aboveground biomass across niche, *TBGB* total belowground biomass, *RB* Riverine bamboo, *WL* woodlots and *HS* Homestead

### Carbon stocks and sequestration potential of the highland bamboo forests

Results showed that the mean biomass carbon of the bamboo forests ranged from 87.52 to 111.56 Mg C ha^−1^ in the entire niches. The highest carbon stock (111.56 Mg ha^−1^) was found in the homestead niche while the smallest amount was recorded in the riverbank niche (87.52 Mg ha^−1^). A total of 21.55% more carbon stocks were accumulated in the homestead niche than in the riverbank niche. However, the carbon stock potential of the homestead and woodlot niches didn’t show a significant difference at p < 0.05 (Table 4).

**Table 4:** Carbon stock and CO_2_ sequestration potential of highland bamboo forests among plantation niches

Niche	TB (Mg ha^−1^)	CS (Mg ha^−1^)	CO_2_ seq. (ha^−1^)
RB	186.22 ± 9.40^b^	87.52 ± 4.42^b^	321.21 ± 16.21
WL	223.35 ± 5.32^a^	104.97 ± 2.50 a	385.25 ± 9.17
HS	237.36 ± 9.46^a^	111.56 ± 4.45 a	409.42 ± 16.33

Different letter indicates significant difference while similar letter showed no significant difference of biomass and carbon stocks among niches at p < 0.05

*TB* Total biomass, *CS* carbon stocks

## Discussion

### Stand structure of highland bamboo forests in the study area

Results show that highland bamboo plantations of the present study area have significant variations in both clump and culm density. The homestead niche had the highest culm density (27,945 culm ha^−1^), while the river basin niche had the lowest (20,375 culm ha^−1^) density of culm. Conversely, the lowest clump density (1,562 clumps ha^−1^) was found in the homestead while the highest density of clump (1,885 ha^−1^) was recorded in the woodlot plantation. The highest culm density in the homestead niche is attributed to better management of bamboo stands as they grow in farmers’ vicinity.

Compared to the present study, a much lower density of 4374 culms ha^−1^ from Cameron [61], and 6267 culms ha^−1^ from Ghana was reported for the lowland bamboo (*O. abyssinica)* [27], while 8840 culms ha^−1^ was reported for highland bamboo (*O. alpina*) from Ethiopia [59]. Comparable densities of 20,467 culms ha^−1^ were reported by Mulatu and Fetene [48] and 20,748 culms ha^−1^ Nigatu et al. [49] for highland bamboo in Ethiopia. However, higher density of culms is reported for the *S. dullooa* (32,376 culm ha^−1^), *P. polymorphum* (43,000 culm ha^−1^) and *M. baccifera* (39,075 culm ha^−1^) from India [57]. Variations in bamboo stand density can be attributed to species differences, tending operations, harvesting intensity, and site conditions.

### Biomass storage among age class and plantation niches

Results indicate that the matured bamboo culms (3–4-year-old) contributed the highest biomass, while old culms (5–6-year-old) stored the lowest biomass. The significant portion of the biomass (> 72%) was found in the young (1–2-year-old) and matured bamboo culms in the three niches. Most of the old culms were harvested in the stands of the studied plantation niches. In contrast, Embaye et al. [59] reported that old culms were dominant in the unmanaged natural bamboo stands of Masha Forest in Ethiopia. In this regard, studies indicate that bamboo management has a greater impact on biomass distribution among age classes, where culms of each age class have an average biomass distribution in well-managed bamboo [62].

The present study found that the total biomass stored by bamboo forests varied across the plantation niches. The homestead niche stored the highest biomass (237.36 Mg ha^−1^) followed by the woodlot (223.35 Mg ha^−1^) and riverbank (186.22 Mg ha^−1^), respectively. The highest biomass storage by the homestead bamboo plantation is due to the application of manure and natural fertilizer, proper harvesting, and management of culms, and protection from illegal harvesting and livestock. The bamboo culms in the woodlot and riverbank niches, on the other hand, are subjected to illegal harvesting and grazing, resulting in reductions in culm density and productivity.

Compared to previous studies, our result is much higher than the reported values of 100 Mg ha^−1^ aboveground biomass [63]; 110 Mg ha^−1^ [59]; 99 Mg ha^−1^ [48], and 108 Mg ha^−1^ [49] for *O. alpina*, and 154.3 – 185.1 Mg ha^−1^ for *O. abyssinica* [2] from Ethiopia. Variations in bamboo biomass storage could be explained by changes in tending operations, harvesting intensity, and site conditions.

### Carbon stock and CO_2_ sequestration potential of highland bamboo forests

The present study found that the total biomass carbon stored by bamboo forests varied across the plantation niches. The total biomass C stored by bamboo forests was the highest in the homestead (111.6 ± 4.4 Mg C ha^−1^), while the riverbank niche stored the lowest amount of carbon (87.5 ± 4.4 Mg C ha^−1^). The total biomass carbon stored by the studied bamboo forests (87.5 ± 4.4 – 111.6 ± 4.5 Mg C ha^−1^) is considerably higher than the reported values of 47 Mg C ha^−1^ [63], 50.76 Mg C ha^−1^ [49], and 51.7 Mg C ha^−1^ [59]. Similarly, lower values of 72.5–87 Mg C ha^−1^ [2] for *O. abyssinica* was reported from Ethiopia.

By and large, the bamboo forests of the study area sequester about 321.21–409.42 tons of CO_2_ eq. in their biomass. Hence, these bamboo forests play a significant role in absorbing a significant amounts of atmospheric CO_2_ and play a great role in mitigating climate change. Bamboo management in all plantation ecosystems would increase the size (diameter) of bamboo culms, accumulating more biomass and sequestering more carbon. Moreover, if harvested bamboo culms are turned into durable products such as permanent construction materials, furniture, art, and so on, bamboo could provide an important and long-term C sink.

In this study, we were unable to develop a model for estimating belowground biomass due to resource constraints. As a result, BGB was calculated using root-to-shoot ratio method [59]. This may under or overestimate the BGB stored by the studied highland bamboo forests. Therefore, allometric models should be developed to precisely estimate the belowground biomass storage capacity of highland bamboo forests.

## Conclusions

This study attests to the carbon stock potential of the highland bamboo forests in the homestead, riverbank and woodlot niches. The highland bamboo forests in the homestead niche stored the highest amount of carbon (111.56 ± 4.45 Mg C ha^−1^), followed by the woodlot (104.97 ± 2.50 Mg C ha^−1^) and the riverbank (87.52 ± 4.42 Mg C ha^−1^) niches, respectively. Generally, the highland bamboo forests of the study area can store a total of 304.05 Mg C ha^−1^ and sequester 1,115.86 tons of CO_2_ eq. in their biomass, playing a critical role in the mitigation of climate change. If bamboo forests are properly managed and harvested culms are turned into durable products, they can sequester more carbon and serve as an important long-term carbon sink.

Bamboos provide income and supports farmers’ livelihoods due to their rapid growth and short harvesting cycle. Furthermore, because bamboo is adaptive to adverse site conditions, it can be planted in degraded lands, which is a common problem in Ethiopian highlands. As a result, expanding bamboo plantations will play critical economic, ecological, and environmental roles in land rehabilitation, soil and water conservation, carbon sequestration, and livelihood improvement.

## Supplementary Information

**Additional file1. **Biometric data of highland bamboo.

## Data Availability

Not applicable.
